# Inferior long-term outcomes after surgery for lumbar disc herniation in patients with prior lumbar spine surgery

**DOI:** 10.1007/s00701-024-05932-3

**Published:** 2024-01-24

**Authors:** Miika Ilvesroiha, Johan Marjamaa, Jari Siironen, Anniina Koski-Palkén

**Affiliations:** 1 Department of Neurosurgery, Helsinki University Hospital, Helsinki, Finland; 2 Faculty of Medicine, University of Helsinki, Helsinki, Finland

**Keywords:** Lumbar disc herniation, Microdiscectomy, Revision surgery, Long-term outcome, Oswestry Disability Index, EuroQol-5D

## Abstract

**Background:**

Previous lumbar spine surgery is a frequent exclusion criterion for studies evaluating lumbar surgery outcomes. In real-life clinical settings, this patient population is important, as a notable proportion of patients evaluated for lumbar spine surgery have undergone prior lumbar surgery already previously. Knowledge about the long-term outcomes after microdiscectomy on patients with previous lumbar surgery and how they compare to those of first-time surgery is lacking.

**Methods:**

The original patient cohort for screening included 615 consecutive patients who underwent surgery for lumbar disc herniation, with a median follow-up time of 18.1 years. Of these patients, 89 (19%) had undergone lumbar spine surgery prior to the index surgery. Propensity score matching (based on age, sex, and follow-up time) was utilized to match two patients without prior surgery with each patient with a previous surgery. The primary outcome measure was the need for further lumbar spine surgery during the follow-up period, and the secondary outcome measures consisted of present-time patient-reported outcome measures (Oswestry Disability Index, EuroQol-5D) and present-time ability to carry out employment.

**Results:**

Patients who received previous lumbar surgeries had a higher need for further surgery (44% vs. 28%, *p* = 0.009) and had a shorter time to further surgery than the propensity score-matched cohort (mean Kaplan–Meier estimate, 15.7 years vs. 19.8 years, *p* = 0.008). Patients with prior surgery reported inferior Oswestry Disability Index scores (13.7 vs. 8.0, *p* = 0.036). and EQ-5D scores (0.77 vs. 0.86, *p* = 0.01). In addition, they had a higher frequency of receiving lumbar spine-related disability pensions than the other patients (12% vs. 1.9%, *p* = 0.01).

**Conclusions:**

Patients with previous lumbar surgery had inferior long-term outcomes compared to patients without prior surgery. However, the vast majority of these patients improved quickly after the index surgery. Furthermore, the difference in the patients’ reported outcomes was small at the long-term follow-up, and they reported high satisfaction with the results of the study surgery. Hence, surgery for these patients should be considered if surgical indications are met, but special care needs must be accounted for when deliberating upon their indications for surgery.

## Introduction

Lumbar disc herniation frequently presents with lower back and radicular pain [9]. Although conservative management is the preferred initial treatment option [21], surgical treatment has become more frequent; the number of lumbar spine surgeries performed rose rapidly in the 1990s [25] and continued to increase in subsequent decades [12, 22]. This trend has been fueled by advances in mini-invasive surgical techniques such as microdiscectomy and endoscopic discectomy [4], as well as positive results from randomized studies showing the efficacy [1, 20, 26] and cost-effectiveness of surgical treatment [23].

However, patients with a prior history of lumbar surgery have been regularly excluded from studies on the treatment effects of surgery for lumbar disc herniation [1, 20, 26]. This is justified on the basis that the inclusion of patients with prior surgery would increase the heterogeneity of the patient cohort. Consequently, there is a lack of studies that report the outcomes for patients with prior lumbar surgery, even though in real-life clinical settings, patients with prior history of lumbar surgery are frequent as 25% of patients undergo further surgery by 10 years [2].

Only a few studies have directly compared the outcome of first-time surgery to revision surgery. A 27-patient series with 41-month follow-up found that the improvement after surgery was similar [19], and in a 16-patient series of contralateral recurrent lumbar disc herniation, the patients with previous surgery had inferior recovery of back pain at six-months but the two-years both groups and similar outcome [7]. However, a larger register study reported that patients with previous surgery have inferior outcome in 12-month follow-up [27]. In our best knowledge, no studies have reported long-term outcomes of microdiscectomy on patients with previous lumbar surgery. However, understanding the long-term outcome is essential, especially when treating younger patients, as they have long lives and working careers ahead of them.

Our previous studies with a young adult patient cohort showed that patients who undergo first-time surgery for lumbar disc herniation have favorable long-term outcomes [14], and their health-related quality of life is comparable to that of the general population nearly two decades after index surgery [15]. As is customary, we excluded patients with previous surgeries from these studies, but here we aim to compare and contrast the results for patients with previous lumbar surgeries to propensity-matched controls without such histories in order to have a more “real-life" understanding of the long-term prognosis for these patients and thus be able to offer better consultation and knowledge to the patients we treat in everyday practice.

## Methods

### Patient cohort

**Fig. 1:**
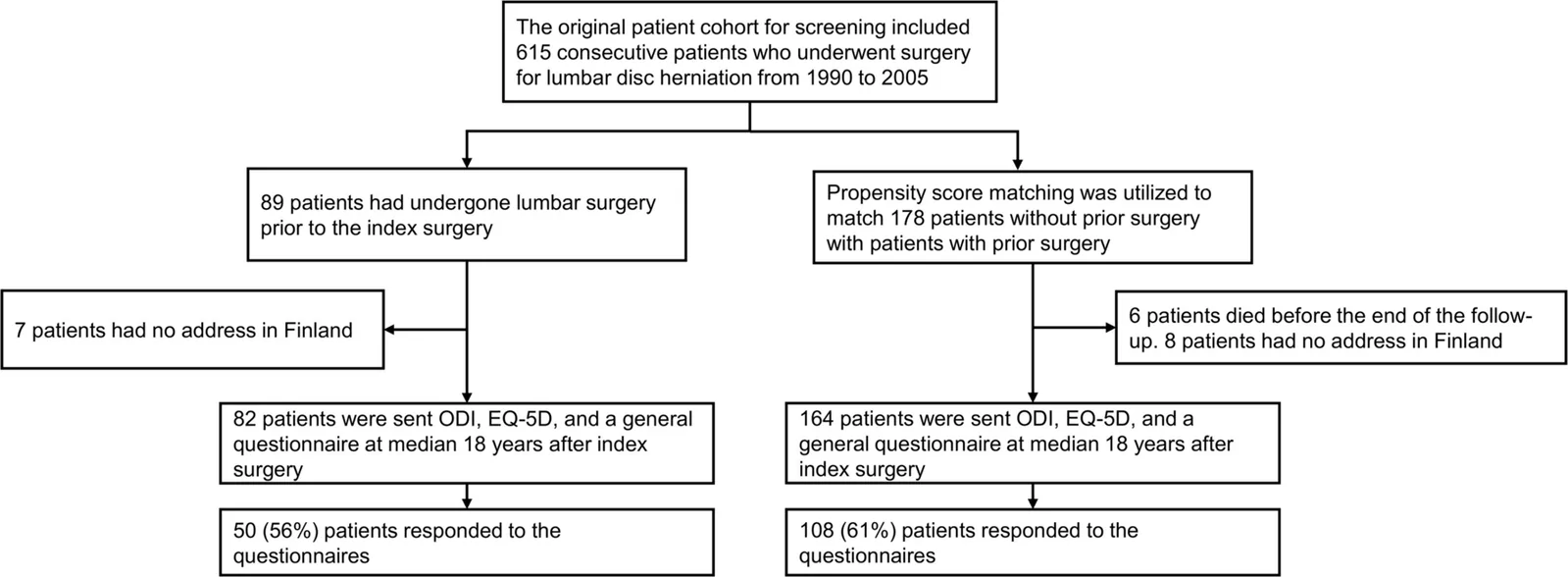
Data gathering and propensity score matching protocol

The original screened patient cohort included 615 consecutive patients who underwent surgery for radiologically confirmed lumbar disc herniation in the Department of Neurosurgery at Helsinki University Hospital from 1990 to 2005. The patients were screened from the registry of all procedures conducted in the Department between 1990 and 2005 by searching for all adult patients under 40-years old with surgery conducted with the diagnose code M51.1 (radiculopathy due to lumbar disc herniation). Additionally, further searches were done with the procedure codes for removal of lumbar disc herniation. A chart review was conducted to confirm eligibility. The inclusion criteria were surgery for a radiologically confirmed lumbar disc herniation, an age between 18 and 40 years at the time of the current surgery, and a history of previous lumbar spine surgery. Patients whose symptoms were caused by conditions other than lumbar disc herniation, such as tumor or trauma, were excluded. Patients with simultaneous lumbar spine stenosis were included if the main indication for the surgery was herniated lumbar disc. For the controls, the inclusion criteria were identical, aside from no previous lumbar spine surgery. The study protocol was approved by the ethics committee of Helsinki University Hospital prior to its initiation and is summarized in Fig. 1.

The standard surgical procedure conducted was microdiscectomy I.e. sequestectomy. The sequesterectomy was performed with a minimally invasive approach. Only the herniated portion of the disc was removed, and removal of the lumbar disc material otherwise was avoided. Bony structures were decompressed only if necessary for obtaining adequate access to the spinal canal or for concurrent microdecompression of spinal stenosis. No patient in this series underwent stabilization by fusion in the index surgery. Types and proportions of surgical techniques used are listed in Table 1.

**Table 1:** Patient baseline characteristics

	Previous surgery cohort	Propensity-matched patient cohort	*p*-value ^1^	All patients without previous surgery	*p*-value^2^
Number of patients	89	178		526	
Age at the time of the surgery (years, median, IQR)	33.9 (7.6)	33.7 (7.3)	0.83	33.2 (7.6)	0.45
Gender			1		0.29
Male	59 (66%)	117 (66%)		314 (60%)	
Female	30 (34%)	61 (34%)		212 (40%)	
Smoker ^3^	18 (31%)	46 (37%)	0.51	133 (36%)	0.55
Body mass index^4^					
Mean (SD)	25.5 (4.2)	24.7 (3.3)	0.15	24.5 (3.5)	0.044
< 18.5	0 (0%)	0 (0%)		6 (1.2%)	
18.5–24.9	46 (55%)	95 (57%)		291 (58%)	
25–30	25 (30%)	63 (38%)		172 (34%)	
30 +	13 (16%)	9 (5.4%)		31 (6.2%)	
Duration of symptoms preoperatively			0.23		0.24
Under 6 months	51 (57%)	96 (54%)		292 (56%)	
6 to 12 months	17 (19%)	50 (28%)		139 (26%)	
Over 12 months	21 (24%)	32 (18%)		95 (18%)	
Previous lumbar surgery			-		−
0	0 (0)	−		−	
1	72 (81%)	−		−	
2	14 (16%)	−		−	
3 or more	3 (3%)	−		−	
Previous surgery on same intervertebral level	69 (78%)	−		−	
Time from previous surgery (years, median, IQR)	2.7 (4.6)	−		−	
Index surgery technique			0.88		0.94
Microdiscectomy	86 (97%)	170 (96%)		503 (96%)	
Microdiscectomy and microdecompression	2 (2.3%)	6 (3.3%)		16 (3.0%)	
Only microdecompression	1 (1.1%)	2 (1.1%)		7 (1.3%)	
Median follow-up period (years, IQR)	18.6 (5.7)	18.1 (5.4)	0.88	18.3 (6.5)	0.44
Age at the end of the follow-up (years, median, IQR)	53.7 (11.4)	53.6 (9.2)	0.84	53.0 (8.9)	0.83

^1^Difference between the propensity score-matched patient cohort without previous surgery and the patient cohort with previous surgery

^2^Difference between the patient cohort with previous surgery and the patients without previous surgery

^3^Information on preoperative smoking was available for 429 patients

^4^Weight and height were available for 584 patients

### Data collection

Patient baseline characteristics, such as age, gender, body mass index at the time of the index surgery, symptoms prior to surgery, and history of previous lumbar surgery, were acquired from medical records. The surgical reports were evaluated to record the duration of the surgery and possibly the surgical complications. The records of the hospital stay and the discharge report were accessed to evaluate short-term improvement. The improvement was retrospectively evaluated with a 5-point Likert scale (5 = substantially improved, 4 = slightly improved, 3 = the same, 2 = slightly declined, 1 = substantially declined) [17]. The routine protocol at the time was to schedule a clinical follow-up visit for the patients at two to three months post-surgery. We used these reports to record the symptoms that persisted after surgery and to determine the clinical improvement score. The remainder of the patients’ medical records at Helsinki University Hospital were thoroughly examined to record any possible further lumbar surgeries during the follow-up period.

Afterwards, all patients who were presently living in Finland were sent a letter containing a consent form for participating in the questionnaire part of the study, as well as a general questionnaire on lifetime lumbar surgeries, satisfaction with surgery, and employment status. The questionnaire also included the Oswestry Disability Index (ODI) [10] and the EuroQol-5D (EQ-5D) survey [5]. A reminder letter was sent once to the patients who did not reply within three months.

### Propensity score matching

To decrease the difference in baseline characteristics between the patient cohort with previous surgery and the cohort without such procedures, propensity score matching was used [3]. The propensity score matching was conducted with the R package Matchit [13]. The method used was nearest neighbor matching, with a 2:1 ratio of patients without previous surgery to patients with previous surgery. The matched preoperative characteristics were age at the time of the index surgery, sex, and duration of the follow-up period.

The propensity score-matched patient cohort without previous surgery (PSM cohort) was compared to the rest of the patients in the originally-screened cohort without previous surgery to confirm that there was no significant difference between these groups. An analysis was conducted on the following variables: reoperation during the first 30 days, further surgery after 30 days, time to further surgery, lumbar fusion, spinal cord stimulator, symptom relief at discharge and at follow-up clinical visit, EQ-5D scores, and ODI scores.

### Statistical methods

All statistical analyses were conducted using R (version 4.0.1). The analyses were done by comparing patients with previous surgeries to the propensity score-matched patient cohort who did not receive such treatment. Continuous variables were analyzed with t-tests, and ANOVA was performed if there were more than two dependent groups. The Levene test was run prior to the t-test to select the correct assumption of equality of variance. Ordinal variables were analyzed with the Wilcoxon rank sum test or Kruskal–Wallis test, depending on the number of dependent groups. Time to further surgery analyses were conducted with Kaplan–Meier estimates, and the difference between times to surgery was evaluated with the log-rank test.

## Results

### Patient demographics

The screened patient cohort included 615 patients, of whom 89 (18%) had undergone lumbar spine surgery prior to the index surgery (Table 1). Seventy-two patients had received one previous lumbar surgery, 14 patients had received two, and three patients had undergone over two prior surgeries. The type of the surgical technique for previous surgery was unknown for 44% of patients. This was due to the patients who underwent the prior surgery at different hospital district or private clinic, and the surgical report was unavailable. Of the cases in which the type of the surgery could be identified, 53% had been microdiscectomies, 26% open discectomies (without microscope), 15% nucleotomies, 4% explorations, and 2% microdecompressions. The median time from previous surgery was 2.7 years (IQR 4.6), and in 78% of the cases, the patient had previously undergone surgery at the same intervertebral level.

Before the propensity score matching, the patients in the cohort with previous surgery were older (median 33.9 vs. 33.2), were less frequently female (34% vs. 40%), and had higher BMIs (mean 25.5 vs. 24.5) compared to all patients without previous surgery. After matching, the characteristics were well balanced (Table 1).

The index surgeries conducted were similar for both patient groups in the propensity score matched patient cohort. Of the patients with previous surgery, 97% underwent microdiscectomy, 2% microdiscectomy and microdecompression, and 1% solely microdecompression. For the patients without previous surgery, the percentages were 96%, 3%, and 1%.

### Short-term recovery after surgery

**Fig. 2:**
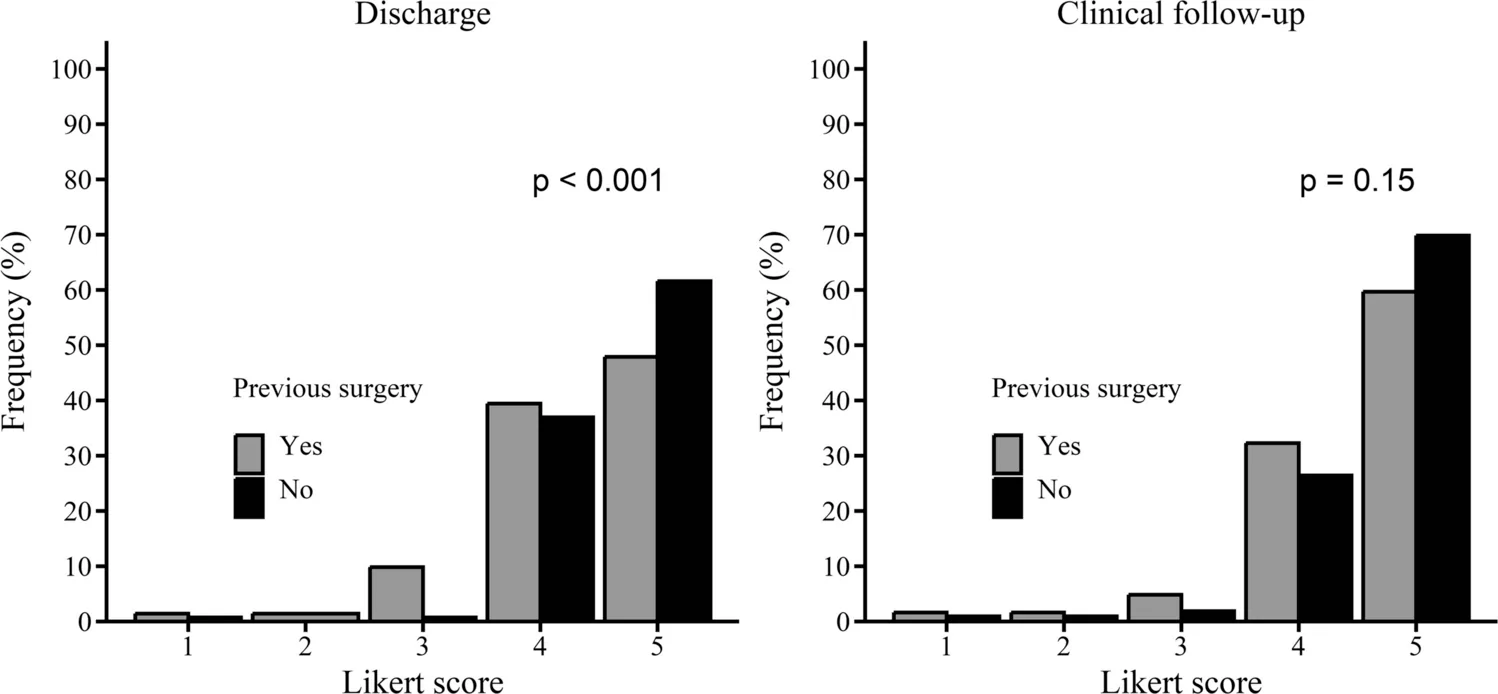
Likert scores at discharge and at the short-term clinical follow-up visit for patients with previous lumbar surgery and propensity-matched control patients without prior surgery (Rating scale: 5 = substantially improved, 4 = slightly improved, 3 = the same, 2 = slightly declined, 1 = substantially declined). The plot on the left shows the scores at discharge (*n* = 209), and the plot on the right shows the scores at the clinical follow-up at a median of 50 days after surgery (*n* = 168)

**Fig. 3:**
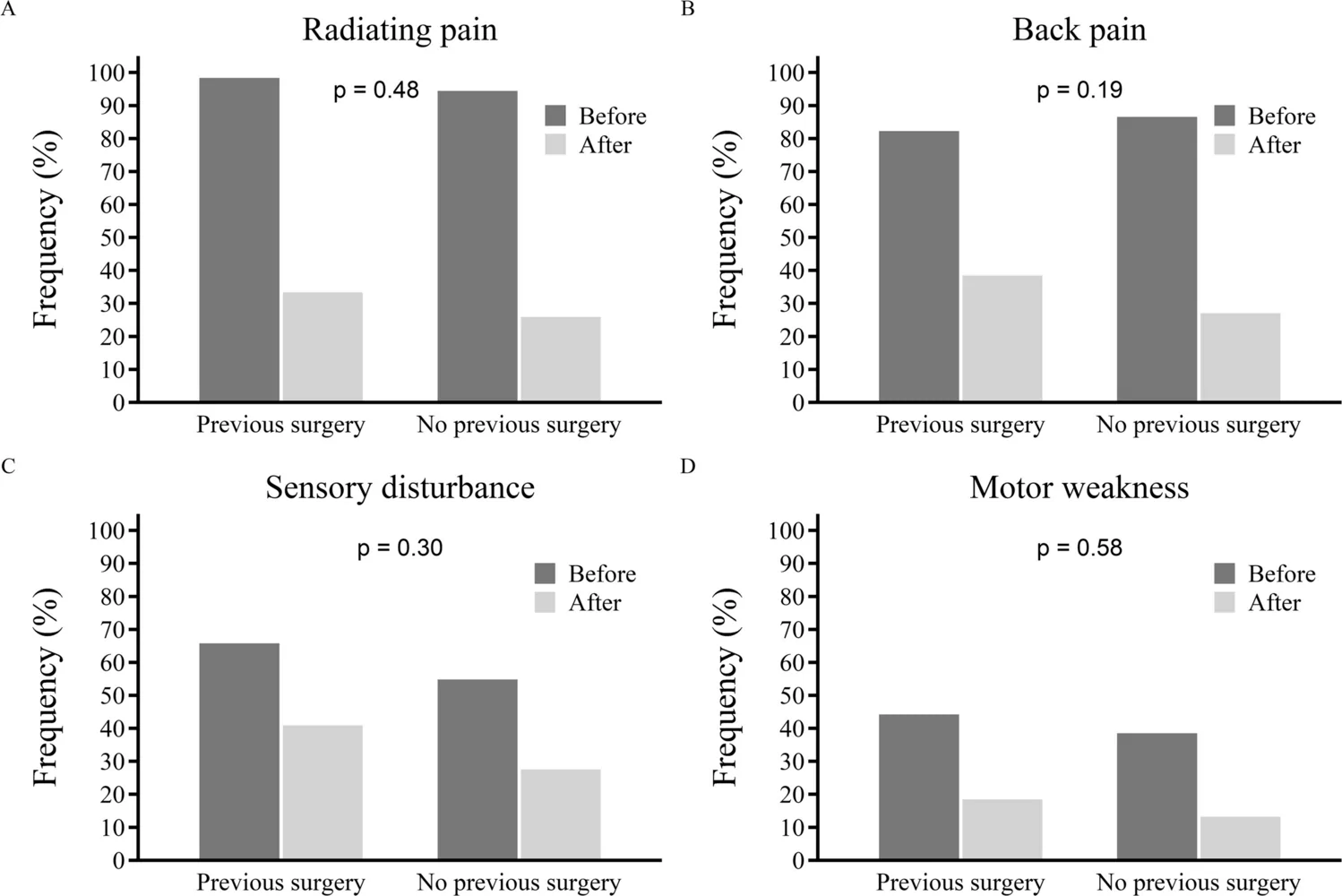
Rate of symptom relief after surgery at the short-term clinical follow-up at a median of 50 days after surgery. Figures **A**, **B**, **C**, and **D** present the relief of back pain, radiating leg pain, sensory disturbance, and motor weakness. The p-values show the differences in the resolution of symptoms between the patients with previous surgery and those without it

The overall improvement evaluated with a 5-point Likert scale showed that at discharge, 87% of patients with a previous surgery experienced improvement in their clinical conditions as measured on the Likert scale; however, they had less improvement compared to the propensity score-matched control cohort (p < 0.001). Nevertheless, the patients with previous surgery continued to improve, and at the clinical follow-up (median of 50 days after surgery), 92% of patients with previous surgery had improved, and there was no difference between the groups (*p* = 0.15) (Fig. 2). Similarly, as stratified according to symptom type, patients in both cohorts had significant reductions in back pain, leg pain, sensory disturbances, and motor weakness symptoms by the time of the clinical follow-up visit. There were no significant differences between the groups for the resolution of back pain (60% vs. 72%, *p* = 0.19 between cohorts), radiating leg pain (66% vs. 72%, *p* = 0.48), sensory disturbance (59% vs. 48%, *p* = 0.30), or motor weakness (65% vs. 55%, *p* = 0.58) (Fig. 3).

### Further surgeries and time for further surgery

**Fig. 4:**
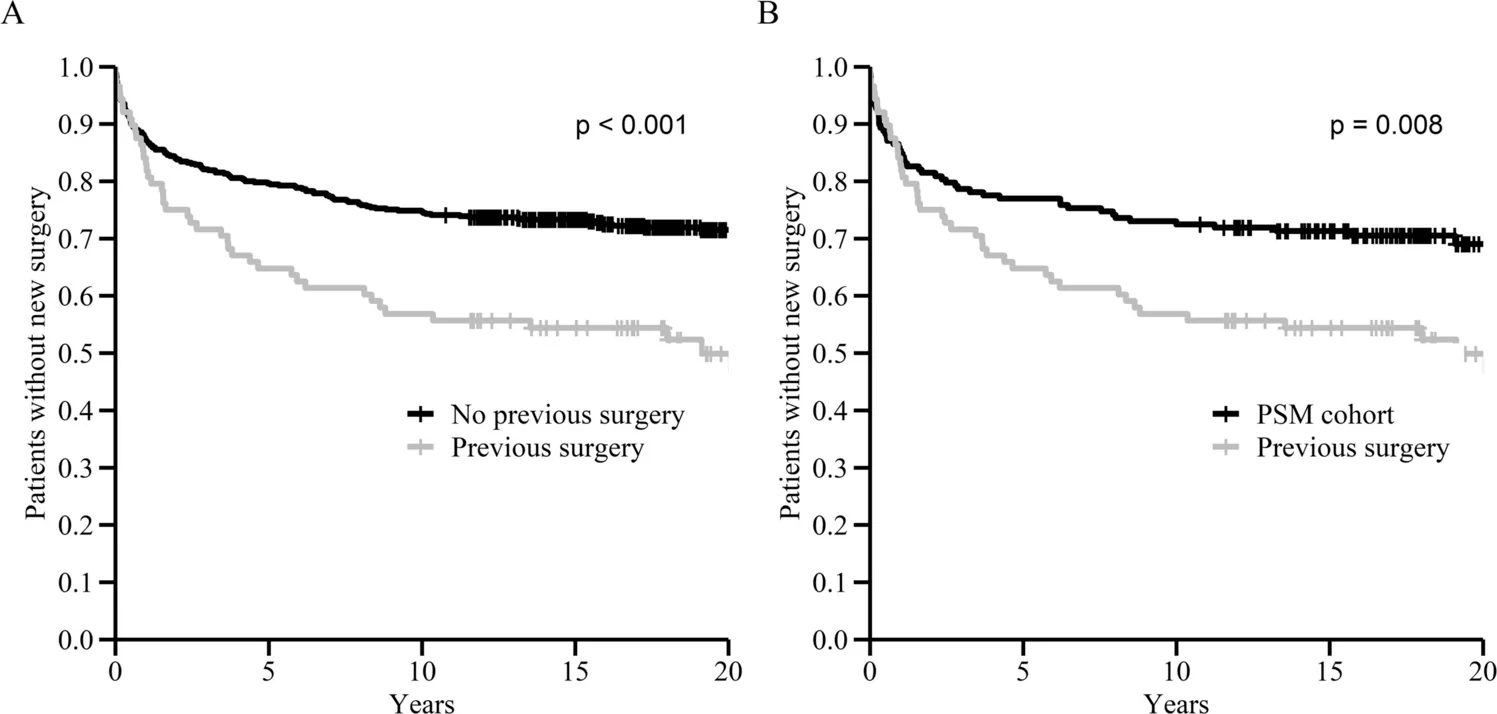
Time to further lumbar surgery for patients with previous lumbar surgery and patients without such procedures. **A**) All patients without previous lumbar surgery (blue, *n* = 525) vs. patients with previous lumbar surgery (red, *n* = 89). **B**) Propensity score-matched patient cohort without previous lumbar surgery (blue, *n* = 178) vs. patients with previous lumbar surgery (red, *n* = 89)

During the first 30 days after the index surgery, 3 patients (3.4%) with previous surgery prior to the index surgery underwent reoperation (Table 2) due to residual or re-herniation of the disc. The frequency of these early revisions was similar in the propensity score-matched cohort (5.1%, *p* = 0.76). After excluding these very early revision surgeries, 39 patients (44%) in the patient cohort with previous surgery underwent further lumbar surgery during the follow-up period, while 49 patients (28%) in the PSM cohort underwent further lumbar surgery (*p* = 0.009 between cohorts). Furthermore, patients with prior lumbar surgery had a shorter time to further surgery, with a Kaplan–Meier estimate of 15.7 years versus 19.8 years in the propensity-matched controls (*p* = 0.008) (Fig. 4). Additionally, the patients with previous surgery underwent more extensive surgeries than the control cohort, as there was a trend of more lumbar fusion procedures (7.8% vs. 3.4%, *p* = 0.13) and significantly more spinal cord stimulator installments (6.7% vs. 0.6%, *p* = 0.006) during the follow-up period (Table 2). The results were similar when comparisons were made between the patients with previous surgery and all of the patients without previous surgery, including the patients in the propensity score-matched control cohort and those not included in it (Table 2).

**Table 2:** Further surgeries during the follow-up period

	Previous surgery	Propensity-matched patient cohort	*p*-value^1^	All patients without previous surgery	*p*-value^2^
Number of patients	89	178		526	
Reoperation within 30 days	3 (3.4%)	9 (5.1%)	0.76	21 (4.0%)	1
Another lumbar surgery during follow-up period^3^	39 (44%)	49 (28%)	0.009	134 (26%)	< 0.001
Same level as index surgery^4^	28 (70%)	44 (80%)	0.33	111 (76%)	0.41
Number of lumbar spine operations after index surgery			0.01		0.001
0	45 (51%)	123 (69%)		376 (72%)	
1	24 (27%)	38 (21%)		107 (20%)	
2	12 (14%)	9 (5.1%)		26 (4.9%)	
3	6 (6.7%)	4 (2.3%)		9 (1.7%)	
4	1 (1.1%)	2 (1.1%)		5 (1.0%)	
5 or more	1 (1.1%)	2 (1.1%)		3 (0.6%)	
Lumbar fusion surgery during follow-up	7 (7.9%)	6 (3.4%)	0.13	18 (3.4%)	0.074
Spinal cord stimulator installment during follow-up	6 (6.7%)	1 (0.6%)	0.006	6 (1.1%)	0.003

^1^Difference between the propensity score-matched patient cohort without previous surgery and the patient cohort with previous surgery

^2^Difference between the patient cohort with previous surgery and the patients without previous surgery

^3^Excluding surgeries done during first 30 days after index surgery and spinal cord stimulator installments

^4^Excluded lumbar surgeries for which the level was unknown

### Oswestry disability index and EuroQol-5D

**Fig. 5:**
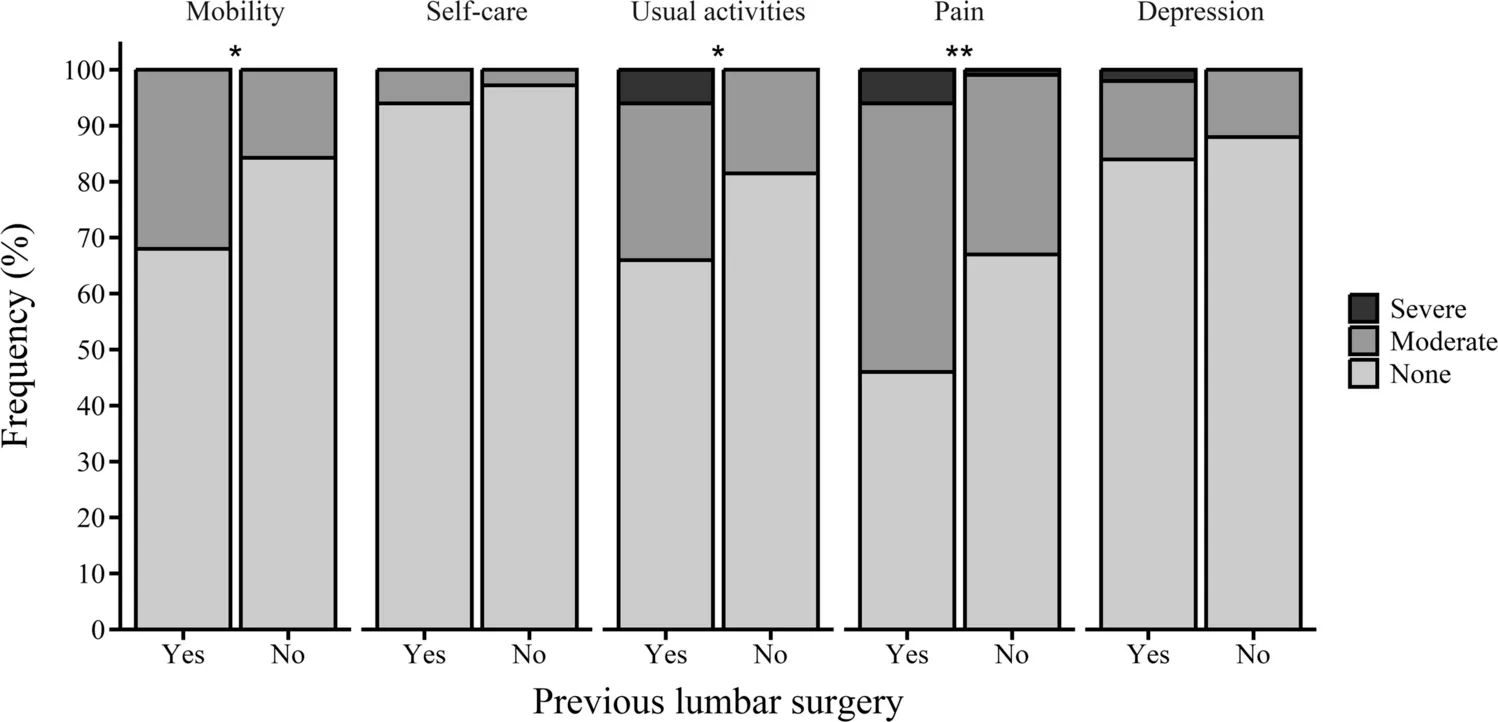
EQ-5D answers for patients with and without previous lumbar surgery. The plot shows the answers for patients with previous lumbar surgery (*n* = 50) and for the propensity score-matched patient cohort without previous lumbar surgery (*n* = 108). * < 0.05. ** < 0.01

Patients were sent questionnaires after a median follow-up of 18 years. The patients with prior surgery reported higher ODI scores than the propensity-matched control patients without previous surgeries (13.7 vs. 8.0, *p* = 0.036). Patients with previous surgery also reported lower EQ-5D index scores than the controls (0.77 vs. 0.86, *p* = 0.01). In the EQ-5D dimension analysis, the patients with previous surgery reported more problems in the mobility dimension (*p* = 0.02), as well as in the usual activities (*p* = 0.02) and pain dimensions (*p* = 0.008) (Fig. 5). There was no significant difference in the self-care (*p* = 0.33) or depression dimensions (*p* = 0.47). Additionally, there was a trend toward patients with previous surgery reporting a lower self-perceived quality of life on the EQ-VAS instrument (75.7 vs. 81.7, *p* = 0.092) (Fig. 5).

### Working status, disability pensions, and satisfaction with surgery

**Fig. 6:**
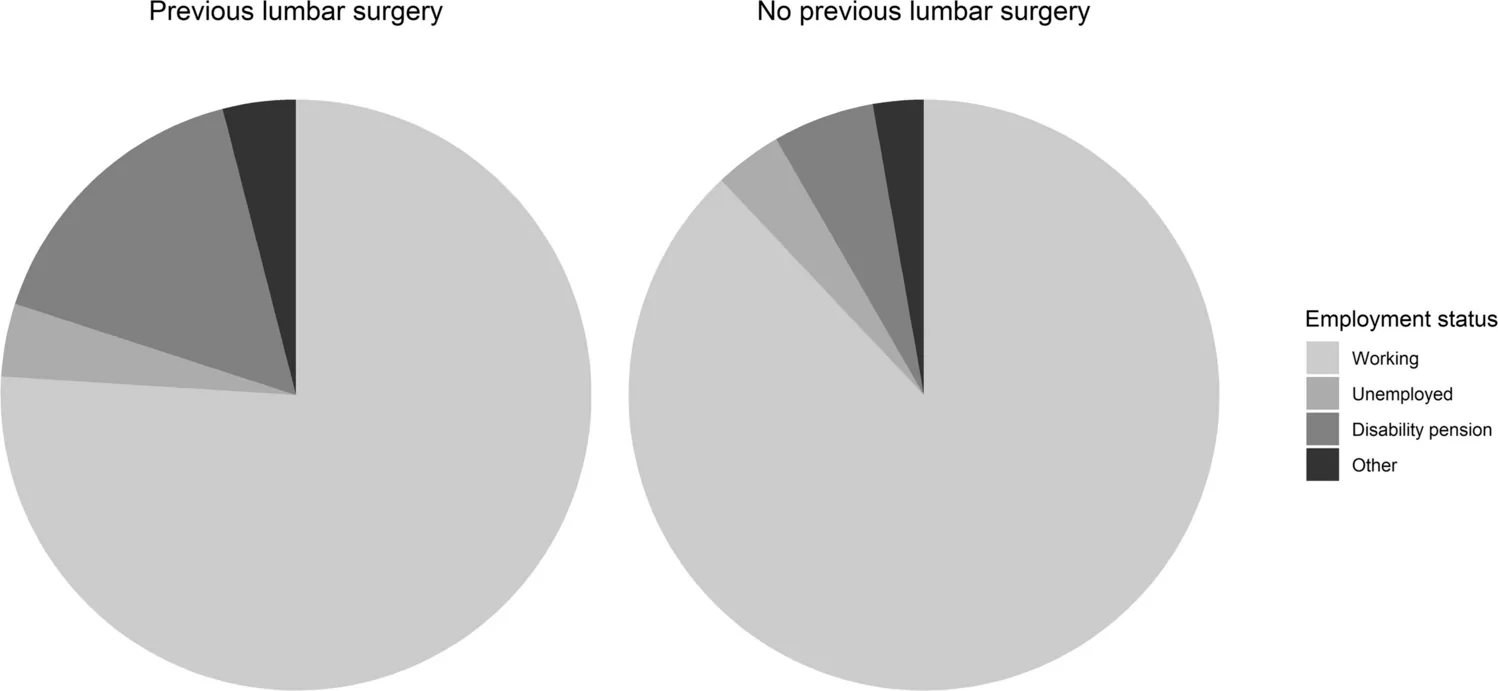
Comparison of employment status between the patients with previous lumbar surgery and the propensity score-matched patient cohort without previous lumbar surgery at a median follow-up of 18 years after index surgery, presented as percentage (%) of patients who responded to questionnaires (*n* = 158)

The number of responding patients with previous lumbar surgery on disability pension was eight out of 50 respondents (16%), while the disability pension rate was 5.6% for the PSM cohort without previous lumbar surgery (Fig. 6). Patients with previous surgery had a higher frequency of lumbar spine-related disability pensions than the PSM cohort (12% vs. 1.9%, *p* = 0.01). Overall, patients with prior surgery had a non-significantly lower employment rate (76% vs. 88%, *p* = 0.064) (Fig. 6).

However, of the patients with prior surgery, 88% reported being either very satisfied or somewhat satisfied with the surgery after a median of 18 years. This rate was similar to that reported by patients in the PSM cohort (91%, *p* = 0.44). Additionally, patients with previous surgery indicated that they would choose surgery again at a similar frequency as other patients (92% vs. 94%, *p* = 0.73).

## Discussion

We evaluated the effect of previous lumbar surgery in a cohort of 615 patients who underwent surgery for lumbar disc herniation prior to the age of 40 years. Of these patients, a high percentage (19%) had undergone prior lumbar surgery. This highlights the significance of this patient subpopulation in real-life clinical settings. In this study, we investigated the long-term outcomes of these patients compared to those of patients who had not undergone any previous surgeries. The results showed that the patients with previous surgery had inferior long-term outcomes compared to the other patients in the long term. Specifically, they had a higher need for further lumbar surgery (44% vs. 28%) and a shorter time to the next procedure during the follow-up period of 18 years. Additionally, nearly two decades after the index surgery, while the patients with prior lumbar surgery reported lower ODI scores and EQ-index values, a higher proportion were on disability pensions due to back-related reasons than patients who underwent their first lumbar surgery.

We also observed a higher rate of further lumbar spine surgery for patients with previous surgery during the median 18-year follow-up period. This finding is in accordance with earlier reports indicating that people who had lumbar disc surgery had a ten times higher rate of needing a new surgery for disc herniation than the general population [6], and that patients who had any kind of prior lumbar surgery had a significantly higher number of reoperations after lumbar surgery in a short 4-year follow-up [8]. However, these studies were conducted in the era in which discectomies were conducted without the aid of microscope and were significantly more extensive than done nowadays. Surgical techniques have significantly changed in the past decades, and this study is the first study reporting long-term need for further surgery in patients with previous surgery conducted with the currently used surgical techniques.

In our study, patients with previous surgery also underwent non-significantly more lumbar fusion surgeries (7.9% vs. 3.4%, *p* = 0.074) and significantly more spinal cord stimulator installment procedures (6.7% vs. 0.6%, *p* = 0.006) than the control patients. This could indicate that a small number of patients initially had more severe degenerative disc disorders, leading to additional and more extensive surgeries, or that they did not experience improvement after the first two surgeries and therefore underwent these procedures to aid their situation. It is likely that some patients in this subpopulation suffer from chronic lower back-related pain, and their outcomes are very different from those patients who experience significant relief of symptoms after a second surgery.

The patients with prior surgery reported an increased rate of lower back-related disability compared to other patients (median 18 years after surgery). This was demonstrated with the mean ODI score of 13.7 in the previous surgery group, which was 8.0 for control patients (*p* = 0.036). This result is similar to reports in previous studies that patients who undergo reoperation during the follow-up period report worse ODI scores [11, 16]. However, it has been proposed that ODI scores from 0 to 20 correspond to minimal disability, and that patients with primary back pain have a mean score of 27, and patients with high chronic back pain have a score of 43 [10]; hence, the patients with prior surgery reported relatively low ODI scores. Functional outcomes can also be evaluated through working status. Although the patients with prior lumbar surgery had a higher rate of lumbar spine-related disability pensions (12% vs. 1.9%, *p* = 0.01), 76% of patients with prior surgery reported that they were employed.

Furthermore, the patients with prior surgery had a lower health-related quality of life, as measured by EQ-index scores, than other patients (0.77 vs. 0.86, *p* = 0.01) at 18 years after the index surgery. As the minimal clinically important difference for the index score has been reported to be 0.07 [24], there is a minor clinically significant quality of life difference between these groups. These findings concur with previous studies, with notably shorter follow-up times, which have shown that patients who undergo revision surgery during the follow-up period have a lower health-related quality of life compared to other patients [11, 18].

However, despite the inferior outcome, nearly 90% of patients with previous surgery experienced that their symptoms had improved at discharge after the index surgery. At the long-term follow-up, they reported high satisfaction with surgery, with nine out of ten patients indicating that they would undergo surgery again, even if they knew the result of their index surgery beforehand. These results were similar between the groups and suggest that patients with previous surgery consider that having the option to undergo surgery is important if their symptoms fail to resolve with conservative treatment.

One of the strengths of this study was that we were able to contact every patient who still lived in Finland 18 years after the index surgery. However, the weakness of the study was that although we had excellent success in contacting the patients, 40% did not respond to the questionnaires. This could have introduced bias in the results if the decision to participate in the study was related to the outcome. However, there was no difference in the frequency of responses between patients with previous lumbar surgery and those without.

## Conclusions

In conclusion, we found that patients with a history of previous lumbar surgery had inferior long-term outcomes compared to patients without prior surgery. This was demonstrated by a higher need for further surgery during the follow-up period, slightly lower health-related quality of life scores, and higher ODI scores a median of 18 years after the index surgery. However, the vast majority of patients with prior surgery experienced significant relief of symptoms after surgery, and they were as satisfied with the results of the surgery as the other patients. Furthermore, nine out of ten patients would undergo surgery again if given the choice; hence, surgery for these patients should be considered if a surgical indication is present, even though the expected outcome of the second surgery is slightly inferior to that of the first.

## Data Availability

The datasets generated during and/or analyzed during the current study are available from the corresponding author on reasonable request.

## References

[1] Atlas SJ, Deyo RA, Keller RB, Chapin AM, Patrick DL, Long JM, Singer DE (1996) The Maine Lumbar Spine Study, Part II. 1-year outcomes of surgical and nonsurgical management of sciatica. Spine (Phila Pa 1976) 21(15):1777–86. 10.1097/00007632-199608010-00011 10.1097/00007632-199608010-00011 8855462

[2] Atlas SJ, Keller RB, Wu YA, Deyo RA, Singer DE (2005) Long-term outcomes of surgical and nonsurgical management of sciatica secondary to a lumbar disc herniation: 10 year results from the maine lumbar spine study. Spine (Phila Pa 1976) 30(8):927–35. 10.1097/01.brs.0000158954.68522.2a 10.1097/01.brs.0000158954.68522.2a 15834338

[3] Austin PC (2011) An Introduction to propensity score methods for reducing the effects of confounding in observational studies. Multivar Behav Res 46(3):399–424 10.1080/00273171.2011.568786 PMC3144483 21818162

[4] Blamoutier A (2013) Surgical discectomy for lumbar disc herniation: surgical techniques. Orthop Traumatol Surg Res 99(1 Suppl):S187-96. 10.1016/j.otsr.2012.11.005 23352565 10.1016/j.otsr.2012.11.005

[5] Brooks R (1996) EuroQol: the current state of play. Health Policy 37(1):53–72 10158943 10.1016/0168-8510(96)00822-6

[6] Bruske-Hohlfeld I, Merritt JL, Onofrio BM, Stonnington HH, Offord KP, Bergstralh EJ, Beard CM, Melton LJ 3rd, Kurland LT (1990) Incidence of lumbar disc surgery. A population-based study in Olmsted County, Minnesota, 1950–1979. Spine (Phila Pa 1976) 15(1):31–5 10.1097/00007632-199001000-00009 2326695

[7] Cinotti G, Gumina S, Giannicola G, Postacchini F (1999) Contralateral recurrent lumbar disc herniation. Results of discectomy compared with those in primary herniation. Spine (Phila Pa 1976) 24(8):800–6. 10.1097/00007632-199904150-00012 10.1097/00007632-199904150-00012 10222532

[8] Ciol MA, Deyo RA, Kreuter W, Bigos SJ (1994) Characteristics in medicare beneficiaries associated with reoperation after lumbar spine surgery. Spine (Phila Pa 1976) 19(12):1329–34 10.1097/00007632-199406000-00005 8066512

[9] Deyo RA, Mirza SK (2016) CLINICAL PRACTICE. Herniated Lumbar Intervertebral Disk. N Engl J Med. 374(18):1763–72. 10.1056/NEJMcp1512658 27144851 10.1056/NEJMcp1512658

[10] Fairbank JC, Pynsent PB (2000) The Oswestry disability index. Spine (Phila Pa 1976) 25(22):2940–52; discussion 2952. 10.1097/00007632-200011150-00017 10.1097/00007632-200011150-00017 11074683

[11] Fritzell P, Knutsson B, Sanden B, Strömqvist B, Hägg O (2015) Recurrent versus primary lumbar disc herniation surgery: patient-reported Outcomes in the Swedish Spine Register Swespine. Clin Orthop Relat Res 473(6):1978–84. 10.1007/s11999-014-3596-8 24711131 10.1007/s11999-014-3596-8 PMC4418986

[12] Grotle M, Småstuen MC, Fjeld O, Grøvle L, Helgeland J, Storheim K, Solberg TK, Zwart JA (2019) Lumbar spine surgery across 15 years: trends, complications and reoperations in a longitudinal observational study from Norway. BMJ Open 9(8):e028743. 10.1136/bmjopen-2018-028743 31375617 10.1136/bmjopen-2018-028743 PMC6688683

[13] Ho D, Imai K, King G, Stuart E (2011) MatchIt: nonparametric preprocessing for parametric causal inference. J Stat Softw 42(8)

[14] Ilvesroiha M, Marjamaa J, Siironen J, Koski-Palkén A (2022) Favorable long-term outcome in young adults undergoing surgery for lumbar disc herniation. Acta Neurochir (Wien). 10.1007/s00701-022-05375-8 10.1007/s00701-022-05375-8 PMC9705424 36205789

[15] Ilvesroiha M, Marjamaa J, Siironen J, Koskinen S, Koski-Palkén A (2023) Favorable long-term health-related quality of life after surgery for lumbar disc herniation in young adult patients. Acta Neurochir (Wien) 165(3):797–805. 10.1007/s00701-023-05522-9 10.1007/s00701-023-05522-9 PMC10006264 36805802

[16] Leven D, Passias PG, Errico TJ, Lafage V, Bianco K, Lee A, Lurie JD, Tosteson TD, Zhao W, Spratt KF, Morgan TS, Gerling MC (2015) Risk factors for reoperation in patients treated surgically for intervertebral disc herniation: a subanalysis of eight-year SPORT data. J Bone Joint Surg Am 97(16):1316–25 26290082 10.2106/JBJS.N.01287 PMC5480260

[17] Likert R. A Technique for the Measurement of Attitudes. Archiv Psychol 140:1–55

[18] Lubelski D, Senol N, Silverstein MP, Alvin MD, Benzel EC, Mroz TE, Schlenk R (2015) Quality of life outcomes after revision lumbar discectomy. J Neurosurg Spine 22(2):173–8 10.3171/2014.10.SPINE14359 25478822

[19] Papadopoulos EC, Girardi FP, Sandhu HS, Sama AA, Parvataneni HK, O'Leary PF, Cammisa FP Jr (2006) Outcome of revision discectomies following recurrent lumbar disc herniation. Spine (Phila Pa 1976) 31(13):1473–6. 10.1097/01.brs.0000219872.43318.7a 10.1097/01.brs.0000219872.43318.7a 16741457

[20] Peul WC, van Houwelingen HC, van den Hout WB, Brand R, Eekhof JA, Tans JT, Thomeer RT, Koes BW; Leiden-The Hague Spine Intervention Prognostic Study Group (2007) Surgery versus prolonged conservative treatment for sciatica. N Engl J Med 356(22):2245–56. 10.1056/NEJMoa064039 10.1056/NEJMoa064039 17538084

[21] Schoenfeld AJ, Weiner BK (2010) Treatment of lumbar disc herniation: evidence-based practice. Int J Gen Med 3:209–14. 10.2147/ijgm.s12270 20689695 10.2147/ijgm.s12270 PMC2915533

[22] Sivasubramaniam V, Patel HC, Ozdemir BA, Papadopoulos MC (2015) Trends in hospital admissions and surgical procedures for degenerative lumbar spine disease in England: a 15-year time-series study. BMJ Open 5(12):e009011. 10.1136/bmjopen-2015-009011 26671956 10.1136/bmjopen-2015-009011 PMC4679892

[23] van den Hout WB, Peul WC, Koes BW, Brand R, Kievit J, Thomeer RT; Leiden-The Hague Spine Intervention Prognostic Study Group (2008) Prolonged conservative care versus early surgery in patients with sciatica from lumbar disc herniation: cost utility analysis alongside a randomised controlled trial. BMJ 336(7657):1351–4. 10.1136/bmj.39583.709074.BE 10.1136/bmj.39583.709074.BE PMC2427123 18502912

[24] Walters SJ, Brazier JE (2005) Comparison of the minimally important difference for two health state utility measures: EQ-5D and SF-6D. Qual Life Res 14(6):1523–32. 10.1007/s11136-004-7713-0 10.1007/s11136-004-7713-0 16110932

[25] Weinstein JN, Lurie JD, Olson PR, Bronner KK, Fisher ES (2006) United States' trends and regional variations in lumbar spine surgery: 1992–2003. Spine (Phila Pa 1976) 31(23):2707–2714. 10.1097/01.brs.0000248132.15231.fe 10.1097/01.brs.0000248132.15231.fe PMC2913862 17077740

[26] Weinstein JN, Lurie JD, Tosteson TD, Skinner JS, Hanscom B, Tosteson AN, Herkowitz H, Fischgrund J, Cammisa FP, Albert T, Deyo RA (2006) Surgical vs nonoperative treatment for lumbar disk herniation: the Spine Patient Outcomes Research Trial (SPORT) observational cohort. JAMA 296(20):2451–9. 10.1001/jama.296.20.2451 17119141 10.1001/jama.296.20.2451 PMC2562254

[27] Zehnder P, Aghayev E, Fekete TF, Haschtmann D, Pigott T, Mannion AF (2016) Influence of previous surgery on patient-rated outcome after surgery for degenerative disorders of the lumbar spine. Eur Spine J. 25(8):2553–62. 10.1007/s00586-016-4383-x 26801193 10.1007/s00586-016-4383-x

